# Clinical, molecular and cytogenetic analysis of 46, XX testicular disorder of sex development with *SRY*-positive

**DOI:** 10.1186/1471-2490-14-70

**Published:** 2014-08-28

**Authors:** Qiu-Yue Wu, Na Li, Wei-Wei Li, Tian-Fu Li, Cui Zhang, Ying-Xia Cui, Xin-Yi Xia, Jin-Sheng Zhai

**Affiliations:** 1 Institute of Laboratory Medicine, Jinling Hospital, Nanjing University School of Medicine, 305 East Zhongshan Road, Nanjing 210002, PR China; 2 Department of Health Care, Jinling Hospital, Nanjing University School of Medicine, Nanjing 210002, PR, China

**Keywords:** 46, XX testicular disorder of sex development (DSD), *SRY*-positive, Sexual hormone

## Abstract

**Background:**

To review the possible mechanisms proposed to explain the etiology of 46, XX sex reversal by investigating the clinical characteristics and their relationships with chromosomal karyotype and the *SRY*(sex-determining region Y)gene.

**Methods:**

Five untreated 46, XX patients with *SRY*-positive were referred for infertility. Clinical data were collected, and Karyotype analysis of G-banding in lymphocytes and Fluorescence in situ hybridization (FISH) were performed. Genomic DNA from peripheral blood of the patients using QIAamp DNA Blood Kits was extracted. The three discrete regions, AZFa, AZFb and AZFc, located on the long arm of the Y chromosome, were performed by multiplex PCRs(Polymerase Chain Reaction) amplification. The set of PCR primers for the diagnosis of microdeletion of the AZFa, AZFb and AZFc region included: sY84, sY86, sY127, sY134, sY254, sY255, SRY and ZFX/ZFY.

**Results:**

Our five patients had a lower body height. Physical examination revealed that their testes were small in volume, soft in texture and normal penis. Semen analyses showed azoospermia. All patients had a higher follicle-stimulating hormone(FSH), Luteinizing Hormone(LH) level, lower free testosterone, testosterone level and normal Estradiol, Prolactin level. Karyotype analysis of all patients confirmed 46, XX karyotype, and FISH analysis showed that *SRY* gene were positive and translocated to Xp. Molecular analysis revealed that the *SRY* gene were present, and the AZFa, AZFb and AZFc region were absent.

**Conclusions:**

This study adds cases on the five new 46, XX male individuals with *SRY*-positive and further verifies the view that the presence of *SRY* gene and the absence of major regions in Y chromosome should lead to the expectance of a completely masculinised phenotype, abnormal hormone levels and infertility.

## Background

The 46, XX disorder of sex development (DSD) is a rare form of sex reversal in infertile men, that was first described by la Chapelle et al. in 1964 and occurred 1:20000 in newborn subjects [1]. By 1996, 150 patients with classical XX male syndrome had been reported, and more than 100 cases of this disorder have been discribed between 1996 and 2006 worldwide [2]. Clinical phenotypes about 46, XX DSD have been identified to three groups, including males with normal phenotype, males with genital ambiguities and males with true hermaphrodites [3]. Ovotesticular DSD, which is characterized by the presence of both testicular and ovarian tissue in the gonads of the same individual, and testicular DSD characterized by a full development of both gonads as testes without any evidence of ovarian tissue [4]. Approximately 80% of individuals with 46,XX testicular DSD present after puberty with normal pubic hair and normal penile size, but small testes, and sterility resulting from azoospermia [5].

The sex-determining region Y gene (*SRY*) locating in Y chromosome, plays a major role in encoding a testis determining factor (TDF) [6,7]. About 90% of these patients have Y chromosomal material including the *SRY* gene, that are usually translocated to the distal tip of the short arm of X chromosome or autosomal chromosomes. About 10% 46, XX males are negative for *SRY* gene, which could carry different degrees of masculinization [8,9].

There are several pathogenic mechanisms explaining 46, XX testicular DSD patients: 1. translocation of Y sequences, including the *SRY* gene, to an X chromosome or to an autosome; 2. a mutation in a gene in the testis-determining pathway triggering testis differentiation in *SRY* negative XX males; and 3. a hidden Y chromosome mosaicism limited to the gonad [10].

This study aimed to describing five 46, XX male DSD with *SRY*-positive, investigating the clinical characteristics and their relationships with chromosomal karyotype and the *SRY* gene.

## Methods

### Participant and Clinical data

We collected 5 untreated patients with *SRY*-positive 46, XX, that were referred for infertility. The physical examination included the measurement of height, potential gynecomastia and the inspection of external sex organs. Bilateral volume was calculated as the sum of the volume of both testes. According to guidelines of the World Health Organization, semen analysis was indicated to azoospermia after centrifugation of the ejaculate.

Serum levels of follicle-stimulating hormone (FSH), Luteinizing Hormone (LH), Estradiol, Prolactin, testosterone and free testosterone were assessed.

All procedures used in the study confirmed to the tenets of the Declaration of Helsinki. The Ethics Committee of Jinling Hospital approved the protocols used. All participants have known to participate in the study. Written informed consents were obtained from all participants.

### Karyotype analysis of G-banding in lymphocytes and Fluorescence in situ hybridization (FISH)

Karyotypes were performed on peripheral blood lymphocytes in five patients respectively including 100 metaphase cells by conventional operating techniques. X chromosome, Y chromosome and *SRY* gene was located using FISH with probes of X chromosome centromere, Y chromosome centromere (CEP X with Spectrum Green, CEP Y with Spectrum Orange, Vysis, Downers Grove, IL; item no.32-111051) and *SRY* gene (*SRY* with Orange,Vysis, Downers Grove, IL; item no.30-190079).

### Molecular analysis

Genomic DNA from peripheral blood of the patients using QIAamp DNA Blood Kits was extracted. The three discrete regions, AZFa, AZFb and AZFc, located on the long arm of the Y chromosome, were performed by multiplex PCRs(Polymerase Chain Reaction) amplification. The set of PCR primers for the diagnosis of microdeletion of the AZFa, AZFb and AZFc region included: sY84, sY86, sY127, sY134, sY254, sY255, SRY and ZFX/ZFY.

## Results

Our five patients had a lower body height. Physical examination revealed that their testes were small in volume, soft in texture and normal penis. No potential gynecomastia and congenital hypospadias were seen. And they all described that they had normal sexual function. Semen analyses showed azoospermia. Endocrinological data indicated that the patients had a higher FSH, LH level, lower free testosterone, testosterone level and normal Estradiol, Prolactin level. General characteristics and endocrine hormone levels are shown in Table 1.

**Table 1:** General characteristics and endocrine hormone levels

**Cases**	**Body height (cm)**	**Age at presentation, development of secondary sex (year)**	**Volume of teste (ml)**	**Stretched penile length (cm)**	**Testosterone (nmol/L)**	**Free testosterone (pmol/L)**	**FSH (IU/L)**	**LH (IU/L)**	**Estradiol (pmol/L)**	**Prolactin (mIU/L)**
**1**	165	12	6	9	6.8	27.7	35.5	13.8	112	201
**2**	162	15	3	8	5.4	15.2	29.2	12.9	70	158
**3**	164	14	4	8.5	8.9	29.4	45.9	25.1	98	78
**4**	167	11	9	11	8.4	28.1	33.7	22.3	107	232
**5**	165	12	7	10	7.0	20.5	31.4	19.6	81	167
**Normal ranges**	≥169	12-14	12-20	8-18	9.4-37.0	30.9-147.6	1-7	2-10	0-250	0-400

**Figure 1:**
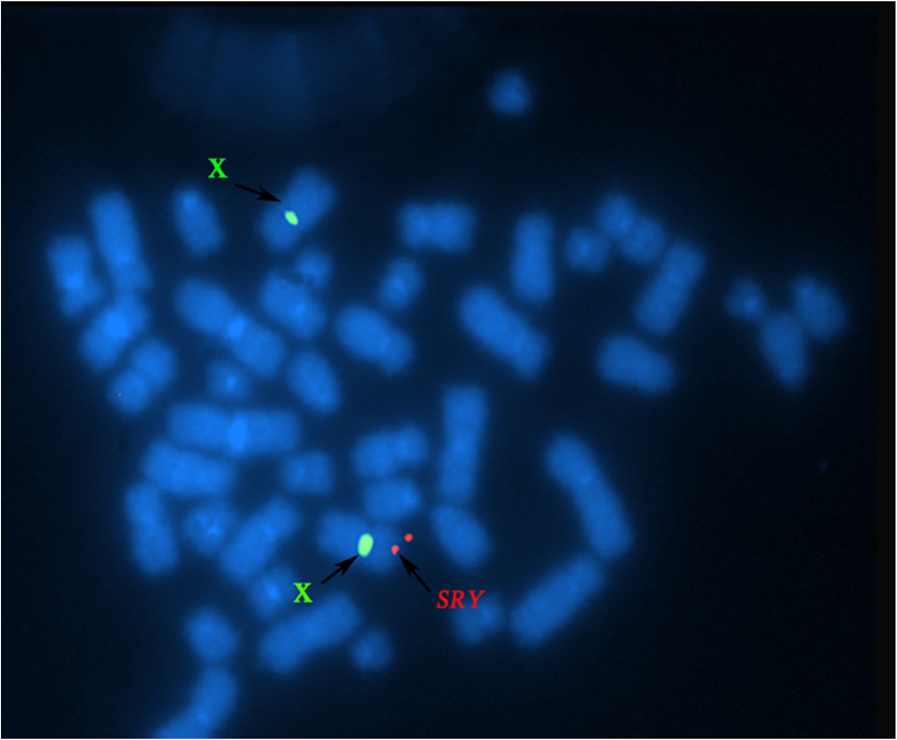
**Fluorescent in situ hybridization (FISH) on metaphase chromosomes of second case with the LSI *SRY*(orange)/CEP X(green) probes.** Metaphase spread showing a normal X chromosome (green signal for centromeric DXZ1 locus) and the *SRY* (orange) translocates to the distal end of short arm of chromosome X.

**Figure 2:**
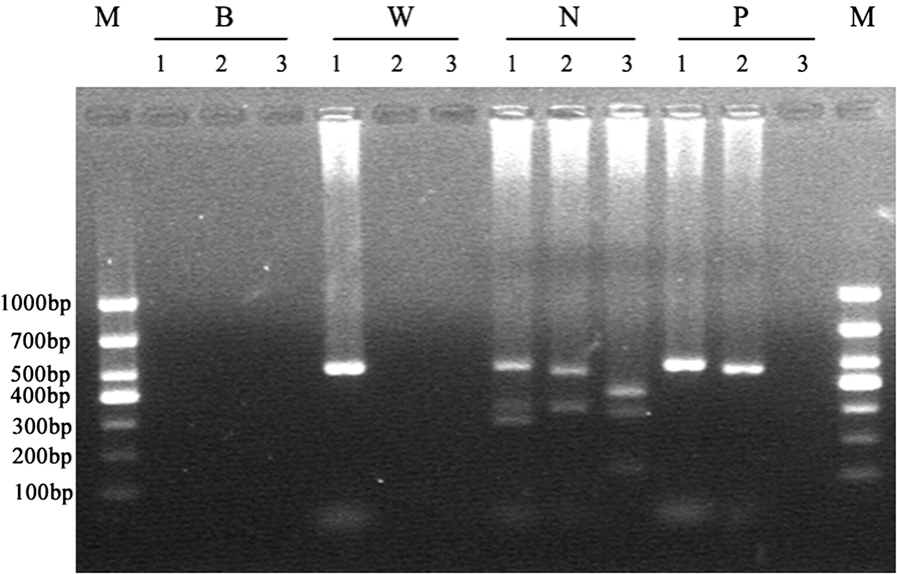
**Result of multiplex polymerase chain reaction (PCR).** Multiplex 1: ZFX/ZFY(690 bp), sY84 (320 bp), sY127 (274 bp); Multiplex 2: SRY (472 bp), sY86 (326 bp); Multiplex 3: sY254 (400 bp), sY134 (301 bp), sY255 (126 bp). M: DL1000 DNAMarker; W: a DNA sample from a woman as a negative control; N: a DNA sample fro-m a normal fertile man as a positive control; P: a DNA sample from the patient; B: a al-ank (water) control.

Karyotype analysis of all patients confirmed 46, XX karyotype, and FISH analysis showed that *SRY* gene were positive and translocated to Xp (Figure 1). Molecular analysis revealed that the *SRY* gene was present, and the AZFa, AZFb and AZFc region were absent (Figure 2).

## Discussion

46, XX male syndrome is a rare sex reversal syndrome characterized by a female karyotype in discordance with a male phenotype. 90% of 46, XX testicular DSD usually have a normal male phenotypic heterogeneity at birth and are diagnosed after puberty on genital ambiguities, or infertility [8]. Our research reported that five patients had a female karyotype but were phenotypically male (46, XX males). They had normal external genitalia and masculinization, but showed azoospermia. That might be that all males were *SRY*-positive, which translocated on the short arm of X chromosome, and absent of the spermatogenic factors encoding gene on Yq, such as AZFa, AZFb and AZFc region in Y chromosome.

*SRY* gene is located in the Y chromosome and encodes a high mobility group(HMG) domain, a conserved motif present in many DNA-binding proteins, which could regulate testicular differentiation [11,12]. SRY protein is expressed in the genital ridge before testis formation, and in the testis during the period of testicular formation early in fetal life, until the development of adult testis [13]. Molecular genetics analysis demonstrated that most 46, XX testicular DSD patients carry *SRY* gene which translocated to X chromosome [14-16]. There was a report that an *SRY* gene fragment translocated from Y chromosome to autosomal chromosome [17]. Some patients showed *SRY* negative, who always had external genital ambiguities and gynecomastia. Despite the fact that *SRY* gene is considered to be the main regulatory factor for testis determination, phenotypic variability showed in 46, XX sex reversed cases cannot be explained only by whether *SRY* gene is presented. And a number of other genes such as SOX9, DAX-1, WT1, WNT4, FGF9 and RSPO1 have been involved in the process of gonadal differentiation [8].

The phenotype of the XX male observed in *SRY* positive 46,XX individuals varies greatly, from normal internal and external male gonads to abnormal secondary sexual characteristics, small testes and hypospadias, to a true hermaphrodite. It has been suggested that the variation in phenotype is primarily dependent on two mechanisms: X chromosome inactivation(XCI) pattern and the amount of Y material including *SRY* gene that has been translocated to the X chromosome [18]. Reviewing the literature, 46,XX males with true hermaphrodites or gonadal ambiguity have a small portion of the Y chromosome material translocated to the X, presumably allowing for XCI spreading and inactivating the *SRY* gene [19]. A normal male phenotype is expected to result from a larger Yp *SRY* bearing fragment being translocated to the X chromosome, where the length of the Yp fragment may protect the *SRY* gene from silencing by the spread of XCI [19]. In our study, all five cases have normal external genitalia and masculinization, which is expected that more Y chromosome material is present on the X, presumably protecting the *SRY* gene from the spread of inactivation. Because of the unavailable in specimens from the five cases to further study, we cannot do more molecular analysis to confirm the above point. Till now, both random and non-random XCI patterns have been reported in 46, XX males with a normal male phenotype [18,20]. It is indicated that the XCI pattern may be not associated with the XX male phenotype.

However, another mechaniam, known as the position effect, has been reported to explain the observed phenotypic differences. The phenotypic differences are dependent on the proximity of the breakpoint to the *SRY* gene as well as the presence or absence of cryptic rearrangements affecting the expression of the *SRY* gene [21]. The rearrangements, may result in transcriptional repression, probably by removing essential regulatory elements or alterations of local chromatin structure [22].

Additionly, phenotypic variability might be associated with variations in genetic polymorphisms and copy number variation of specific genes on the X chromosome, such as NROB1 and TAF7L [23,24].

Classical 46, XX male have normal testosterone level and free testosterone level during adolescence, but may decrease in adulthood, leading to hypergonadotropic hypogonadism [25]. Our cases had normal genitalia and were diagnosed for infertility after puberty. The level of testosterone and free testosterone is deficiency in five patients. In addition, high levels of FSH and LH are observed. This might explain that even though the 46, XX male have a normal external genitalia and masculinization, and they are lack of spermatogenesis.

The body heights of the patients we reported were all under 169 cm (the average height of Chinese male) and close to that of normal females. There are some phenotypic similarities between 46,XX men and those with Klinefelter syndrome, but 46,XX men tend to be shorter than men with KS [9]. Kirsch *et al.* indicated that the Y chromosome growth-control gene(*GCY*) which next to the centromere had a possible impact on growth [26]. And there were some papers indicating that *SHOX* gene(short stature homeobox) expression and *SHOX* enhancer regions played a role in the growth [27]. It has been suggested that specific growth genes in the Y chromosome cannot switched to the patients, which might make them to show a female stature. GH (growth hormone) therapy may have some statural effects in the SHOX haploinsufficiency and may be insufficient to prevent the development of skeletal lesions after puberty [28].

## Conclusions

Our reports adds cases on the five new 46, XX male individuals with sex reversal and further verifies the view that the presence of *SRY* gene and the absence of major regions in Y chromosome should lead to the expectance of a completely masculinised phenotype, abnormal hormone levels and infertility.

## Abbreviations

DSD: Disorder of sex development; *SRY*: Sex-determining region Y gene; TDF: testis determining factor; FISH: Fluorescence in situ hybridization; FSH: Follicle-stimulating hormone; LH: Luteinizing Hormone; PCR: Polymerase Chain Reaction; HMG: High mobility group; *SOX9*: SRY (sex determining region Y)-box 9 gene; *WT1*: Wilms tumor 1 gene, *WNT4*, wingless-type MMTV integration site family, member 4 gene, *FGF9*, fibroblast growth factor 9 gene; *RSPO1*: R-spondin 1 gene; *GCY*: Growth-control gene; *SHOX*: Short stature homeobox; GH: Growth hormone; XCI: X chromosome inactivation; KS: Klinefelter syndrome; *NROB1*(*DAX-1*): Nuclear receptor subfamily 0, group B, member 1 gene; *TAF7L*: TAF7-like RNA polymerase II gene.

## Competing interests

The authors declare that they have no competing interests.

## Authors’ contributions

QW carried out the molecular genetic studies and drafted the manuscript. NL, WL, TL and CZ participated in the laboratory work. YC, JZ and XX conceived of the study, and participated in its design and coordination and helped to draft the manuscript. All authors read and approved the final manuscript.

## Pre-publication history

The pre-publication history for this paper can be accessed here:

http://www.biomedcentral.com/1471-2490/14/70/prepub
